# Persistent meningeal enhancement on MRI in an infant with culture-negative bacterial meningitis: a case report and systematic review of the literature (2014–2025)

**DOI:** 10.3389/fped.2026.1799852

**Published:** 2026-07-14

**Authors:** Qunyan Su, Yiping Shao, Jingjing Jin, Yinghua Yan, Jiangyin Sheng, Anqian Tao, Guiying Ruan, Licheng Cui, Yandan Yin

**Affiliations:** Department of Children’s Internal Medicine, Taizhou Maternal and Child Health Hospital, Taizhou City, China

**Keywords:** culture-negative bacterial meningitis, infant, magnetic resonance imaging (MRI), persistent meningeal enhancement, prognosis

## Abstract

**Background:**

Bacterial meningitis (BM) in infants may be complicated by persistent meningeal enhancement on magnetic resonance imaging (MRI) after clinical cure—a phenomenon frequently misdiagnosed as infection recurrence. However, long-term follow-up data on asymptomatic persistent meningeal enhancement in young infants remain scarce. Here, we report a rare case of 5-month persistent meningeal enhancement following culture-negative bacterial meningitis (CNBM) in a 70-day-old infant, and systematically reviewed 17 eligible studies on meningeal enhancement in infantile bacterial meningitis published between 2014 and 2025 to provide comprehensive evidence for clinical practice.

**Case presentation:**

A 70-day-old male infant was admitted on March 22, 2025, with a 2-day history of fever. Laboratory findings were consistent with severe bacterial infection (C-reactive protein [CRP]: 116.16 mg/L, procalcitonin [PCT]: 0.535 ng/mL), and lumbar puncture confirmed central nervous system infection (cerebrospinal fluid [CSF] nucleated cell count: 376.0 × 10^6^/L, 76% neutrophils, protein: 0.86 g/L). Following standardized antimicrobial therapy with meropenem plus vancomycin (total 18-day course) and supportive care, the infant's clinical symptoms, CSF parameters, and systemic inflammatory markers normalized completely. However, contrast-enhanced cranial MRI demonstrated persistent meningeal enhancement for approximately 5 months (from March 27, 2025, to August 26, 2025), without evidence of cerebral parenchymal injury, hydrocephalus, or subdural effusion. Neurodevelopmental assessment using the Gesell Developmental Schedules revealed age-appropriate performance across gross motor, fine motor, language, and social domains, with no neurological sequelae.

**Conclusion:**

Asymptomatic persistent meningeal enhancement after clinically diagnosed CNBM in young infants may represent a benign manifestation of delayed post-inflammatory repair when alternative etiologies and active infection have been reasonably excluded. Clinical decision-making should rely on comprehensive integration of clinical symptoms, laboratory parameters, and imaging findings to prevent overtreatment based solely on isolated MRI abnormalities. Given the diagnostic limitations inherent in culture-negative cases, long-term serial follow-up is essential to confirm the benign prognosis of such imaging findings.

## 1 Introduction

Bacterial meningitis (BM) remains a leading cause of life-threatening central nervous system (CNS) infection in infants globally. While advances in antimicrobial therapy and supportive care have markedly reduced mortality over the past two decades, BM-related neurological complications and long-term sequelae continue to impose substantial clinical and public health burdens (1). Up to 50% of pediatric BM survivors experience acute or delayed complications, including hydrocephalus, subdural effusion, sensorineural hearing loss, seizures, and neurodevelopmental impairment (2, 3). BM pathogenesis involves pathogen invasion across the blood-brain barrier (BBB) via direct penetration of brain microvascular endothelial cells, followed by robust host inflammatory responses that disrupt BBB integrity and trigger meningeal and parenchymal inflammation (4, 5). Even after complete pathogen clearance, residual local inflammation, altered vascular permeability, and ongoing tissue repair may persist, leading to imaging abnormalities that are discordant with clinical recovery.

Integrated interpretation of clinical, laboratory, and imaging data is central to optimal BM diagnosis and management. A landmark multicenter retrospective cohort of 209 infants <1 year of age established that brain MRI has 92.3%–95.7% specificity and 67.4%–83.5% sensitivity for diagnosing BM, with leptomeningeal enhancement being the most sensitive imaging biomarker (6). Other studies have demonstrated that elevated cerebrospinal fluid (CSF) protein and decreased glucose correlate strongly with MRI enhancement, though CSF cytology, biochemistry, and microbiology remain the diagnostic and therapeutic gold standard (7).

However, most existing research has focused exclusively on the acute phase of the disease. The phenomenon of persistent leptomeningeal enhancement following complete clinical recovery remains poorly characterized (8). While acute-phase enhancement reliably reflects inflammatory severity (7), the prevalence, underlying mechanisms, and clinical significance of post-treatment persistent enhancement remain undefined. This critical knowledge gap frequently leads clinicians to misinterpret isolated persistent enhancement as treatment failure or infection relapse, resulting in unnecessary prolonged antimicrobial courses, repeated invasive procedures, and avoidable patient harm.

Here, we report a rare case of culture-negative bacterial meningitis (CNBM) in a 70-day-old infant who achieved complete clinical and CSF resolution following guideline-directed therapy but exhibited isolated persistent leptomeningeal enhancement for more than five months. We also systematically reviewed all eligible studies on meningeal enhancement in infantile BM published between 2014 and 2025. Integrating serial MRI findings, longitudinal clinical follow-up, and pooled literature evidence, this study delineates the clinical characteristics and prognostic patterns of asymptomatic persistent meningeal enhancement after infantile CNBM, explores underlying pathophysiological mechanisms including meningeal fibrosis and delayed BBB repair, and proposes an optimized diagnostic and follow-up framework based on integrated clinical-laboratory-imaging assessment to better align imaging interpretation with true clinical status and prevent overtreatment driven by isolated radiological abnormalities.

## 2 Case presentation

### 2.1 Patient history

A 70-day-old male infant (full-term spontaneous vaginal delivery on January 11, 2025, birth weight 3.0 kg, Apgar score 10; exclusively breastfed, with age-appropriate growth and development, up-to-date on all routine vaccinations; no significant past medical history) presented to our hospital on March 22, 2025, with a 2-day history of fever. His temperature ranged from 38.0℃ to 39.0℃, peaking at 39.4℃, accompanied by moaning and irritability. No cough, wheezing, vomiting, or seizures were reported. At a local hospital, serum C-reactive protein (CRP) was measured and found to be above reference range. Intravenous ceftriaxone was administered at approximately 04:00 on March 22, along with vitamin C. Oral paracetamol was administered without reduction in fever. His symptoms progressed to paroxysmal crying and occasional staring spells. He was transferred to our emergency department and admitted with a preliminary diagnosis of infectious fever and sepsis.

### 2.2 Physical examination

Vital signs on admission: temperature 39.0℃ (tympanic), heart rate 168 beats per minute, respiratory rate 48 breaths per minute, blood pressure 89/46 mmHg, weight 5.1 kg. The infant was alert and responsive. His neck was supple. The anterior fontanelle measured 1.5 cm × 1.5 cm and was slightly bulging and tense. Mild tachypnea and increased work of breathing were present with fever. Lips were pale without cyanosis or retractions. Pharyngeal injection was noted, with a few petechiae on the soft palate and no purulent exudates. Auscultation of the lungs revealed coarse breath sounds without wheezes or rales. Heart sounds were regular, with no murmurs. The abdomen was soft, with no hepatosplenomegaly. A Bacille Calmette-Guérin (BCG) scar was visible. Extremities were warm. Bilateral ankle clonus was present. Capillary refill time was <3 s.

### 2.3 Laboratory investigations

On admission (March 22, 2025), serum inflammatory markers were measured: CRP 116.16 mg/L, white blood cell (WBC) count 14.19 × 10^9^/L with 53.9% neutrophils, and procalcitonin (PCT) 0.535 ng/mL. Following antimicrobial therapy, these markers changed as follows: by March 31, CRP was <0.20 mg/L, and WBC count and neutrophil percentage were within reference ranges. Platelet count showed a transient elevation followed by a decline. Hemoglobin and red blood cell counts increased steadily (Table 1).

**Table 1:** Serial complete blood count and inflammatory markers.

Date (YYYY/MM/DD)	CRP (mg/L)	WBC (×10⁹/L)	Neutrophil (%)	Hb (g/L)	PLT (×10⁹/L)	PCT (ng/mL)
2025/3/22	116.16	14.19	53.9	85	218	0.535
2025/3/23	83.74	18.75	82.5	82	267	0.04
2025/3/24	29.06	15.01	72.3	86	381	–
2025/3/27	3.38	14.66	31.1	91	671	–
2025/3/31	<0.20	7.4	27.9	103	617	–
2025/4/5	1.35	7.49	13	112	389	–
Reference range (70-day-old infants)	<1.0	5.0–15.0	31–40	94–130	150–400	<0.05

Emergency lumbar puncture (LP) was performed on March 22. Cerebrospinal fluid (CSF) was clear and colorless, with a nucleated cell count of 376.0 × 10^6^/L (76% neutrophils), protein 0.86 g/L, glucose 2.9 mmol/L, chloride 120.4 mmol/L, and a positive Pandy test. No bacteria were detected on CSF Gram stain or culture. Concurrent CSF Xpert MTB/RIF, blood T-SPOT.TB, and tuberculin skin test (induration <5 mm) were negative. Blood and CSF (1→3)-β-D-glucan and galactomannan assays were also negative.

Serial LPs were performed on March 26, April 2, April 7, and May 18. Nucleated cell count was 0 × 10^6^/L by March 26. Pandy test was negative by April 7, and protein was 0.40 g/L by April 7. All CSF parameters were within reference ranges at follow-up on May 18. Transient elevation of CSF red blood cells was noted on March 26, April 2, and April 7 (Table 2).

**Table 2:** Serial cerebrospinal fluid (CSF) analysis and culture results.

Date (YYYY/MM/DD)	Nucleated cells (×10^6^/L)	Red blood cells (×10^6^/L)	Glucose (mmol/L)	Protein (g/L)	Chloride (mmol/L)	Pandy test	Culture result
2025/3/22	376	70	2.9	0.86	120.4	+	Negative
2025/3/26	0	2,500	3.1	1.14	124.5	±	Negative
2025/4/2	0	750	2.4	0.51	126.2	±	Negative
2025/4/7	2	1,000	2.4	0.4	123.4	–	Negative
2025/5/18	5	0	2.7	0.37	122.8	–	Negative
Reference range (70-day-old infants)	0–9	0	1.7–3.9	0.2–0.99	110–122	Negative	Negative

Additional admission tests, including serum biochemistry, arterial blood gas analysis, troponin I, coagulation profile, plasma D-dimer, urinalysis, and stool routine, were all within age-appropriate reference ranges. Brainstem auditory evoked potentials were within normal limits. Echocardiography revealed a secundum atrial septal defect. Cardiology follow-up was arranged.

### 2.4 Imaging studies

**Figure 1:**
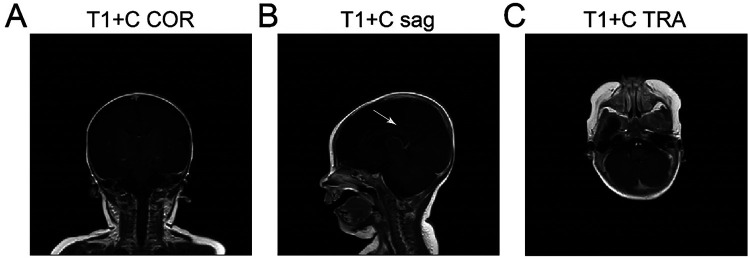
Contrast-enhanced brain MRI on march 27, 2025. **(A)** Coronal T1-weighted postcontrast image. **(B)** Sagittal T1-weighted postcontrast image. **(C)** Axial T1-weighted postcontrast image.

Non-contrast head CT performed on March 22 was unremarkable. Contrast-enhanced brain magnetic resonance imaging (MRI) was performed on March 27 (day 5 of antimicrobial therapy). For neonates with confirmed bacterial meningitis, some experts recommend brain neuroimaging—preferably MRI—toward the end of therapy, and a pediatric review specifically notes MRI 48–72 h before the anticipated end of treatment to detect complications such as cerebritis or abscess (9, 10). MRI revealed diffuse leptomeningeal enhancement. No cerebral infarction, abscess, ventriculitis, or subdural effusion was identified (Figure 1).

**Figure 2:**
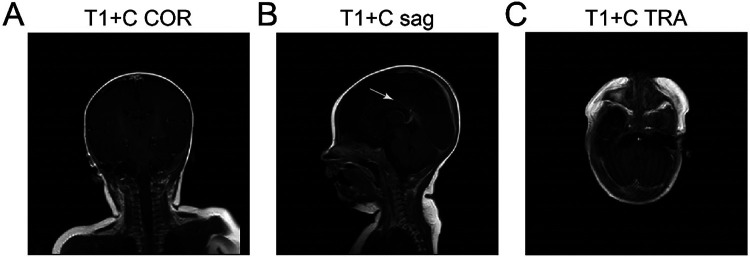
Contrast-enhanced brain MRI on April 6, 2025. **(A)** Coronal T1-weighted postcontrast image. **(B)** Sagittal T1-weighted postcontrast image. **(C)** Axial T1-weighted postcontrast image.

**Figure 3:**
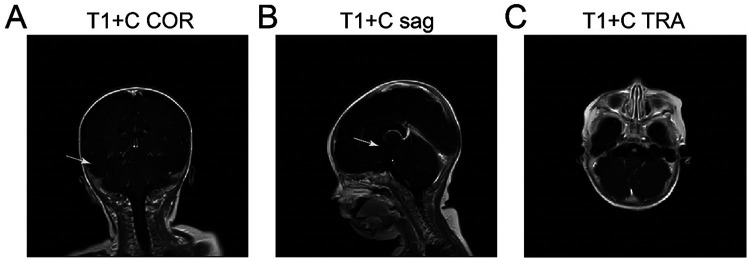
Contrast-enhanced brain MRI on May 16, 2025. **(A)** Coronal T1-weighted postcontrast image. **(B)** Sagittal T1-weighted postcontrast image. **(C)** Axial T1-weighted postcontrast image.

**Figure 4:**
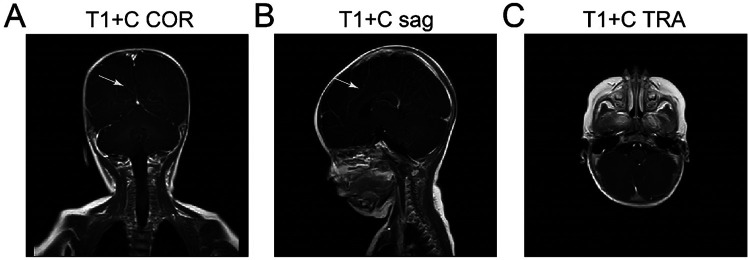
Contrast-enhanced brain MRI on August 26, 2025. **(A)** Coronal T1-weighted postcontrast image. **(B)** Sagittal T1-weighted postcontrast image. **(C)** Axial T1-weighted postcontrast image.

Repeat contrast-enhanced MRI was performed on April 6 (day 15 of therapy). Leptomeningeal enhancement persisted, with no change in degree or extent compared to the prior study (Figure 2). Follow-up MRI on May 16 demonstrated no significant change in leptomeningeal enhancement (Figure 3). A second follow-up MRI on August 26 showed persistent leptomeningeal enhancement, with no new imaging abnormalities (Figure 4).

### 2.5 Treatment course

On admission, empirical antimicrobial therapy was initiated immediately with meropenem 0.075 g intravenously every 8 h. After cerebrospinal fluid (CSF) results were available, the regimen was adjusted to meropenem 0.2 g intravenously every 8 h (March 22 to April 8, 2025) combined with vancomycin 0.075 g intravenously every 6 h (March 22 to March 29, 2025). Adjunctive therapy included dexamethasone 0.15 mg/kg every 6 h (March 22 to March 25, 2025), mannitol 2.5 mL/kg every 6 h (March 22 to March 27, 2025), and supportive care.

### 2.6 Clinical course and follow-up

The infant's condition improved rapidly. His peak fever decreased to below 38.5℃ on March 23, and he became afebrile on March 25. Moaning and irritability resolved completely, and feeding returned to baseline. Vancomycin was discontinued on March 29 after 7 days of therapy. Meropenem was continued as monotherapy until April 8, for a total antimicrobial course of 18 days.

The infant was discharged on April 8. A structured long-term follow-up protocol was established. Two comprehensive follow-up visits were conducted on May 16 and August 26, including assessment of growth parameters, neurodevelopmental evaluation using the Gesell Developmental Schedules, neurological examination, CSF analysis, and contrast-enhanced MRI. At both visits, growth parameters and developmental quotients across gross motor, fine motor, language, and social domains were consistent with age- and sex-matched peers. Neurological examination was unremarkable. CSF routine, biochemistry, and culture were within reference ranges on May 18. Contrast-enhanced MRI at both follow-ups showed persistent leptomeningeal enhancement. No clinical recurrence of infection or new imaging complications was observed. Follow-up contrast-enhanced MRI and neurodevelopmental assessment are planned every 3–6 months.

### 2.7 Systematic review methods

A systematic search of major English and Chinese databases—including PubMed, Embase, Cochrane Library, Web of Science, CNKI, and Wanfang—was conducted for the period January 2014 to December 2025 using the terms “bacterial meningitis” AND “infant” AND “meningeal enhancement” OR “MRI.” Literature screening and exclusion criteria were as follows: (i) Study subjects aged >12 months; (ii) Studies related to non-bacterial meningitis (such as viral, tuberculous, and fungal meningitis); (iii) Studies without reporting meningeal enhancement or cranial MRI imaging data; (iv) Secondary studies including reviews and meta-analyses (only primary studies such as cohort studies, case series, and case reports were included); (v) Studies with incomplete data (lacking core information such as age, pathogen, and prognosis) or duplicate publications. A total of 17 eligible studies were finally included. Detailed search strategies, screening process, and characteristics of included studies are provided in Supplementary Materials.

## 3 Discussion

We report a 70-day-old infant with culture-negative bacterial meningitis (CNBM) who presented with fever, irritability, bulging anterior fontanelle, and bilateral ankle clonus. Laboratory evaluation revealed marked peripheral inflammation and cerebrospinal fluid (CSF) pleocytosis with 76% neutrophils, consistent with classic BM features (11–13). The patient responded rapidly to antimicrobial therapy: clinical symptoms improved within 3 days, fever resolved by day 7, inflammatory markers normalized by day 10, and CSF parameters returned to baseline within 1 month. Notably, serial contrast-enhanced magnetic resonance imaging (MRI) demonstrated persistent leptomeningeal enhancement from illness day 5 through month 5, without evidence of cerebral infarction, abscess, ventriculitis, or parenchymal injury. Longitudinal follow-up confirmed normal growth and neurodevelopment with no cognitive or motor sequelae.

The 2004 Infectious Diseases Society of America (IDSA) guidelines for community-acquired bacterial meningitis recommend blood cultures and lumbar puncture prior to antibiotic administration in suspected cases (12). However, pre-treatment antibiotics significantly reduce CSF culture positivity: studies report negative culture rates of 29% after oral pre-treatment and 44% after parenteral pre-treatment (10). For culture-negative cases with clinical and CSF findings consistent with bacterial infection and excluded alternative etiologies, management principles are identical to culture-positive cases, and a full antimicrobial course is warranted based on clinical judgment (14). Our patient meets the criteria for CNBM. He presented acutely with high fever, moaning, irritability, and focal neurological signs. Markedly elevated inflammatory markers (C-reactive protein [CRP] 116.16 mg/L, procalcitonin [PCT] 0.535 ng/mL) and neutrophilic CSF pleocytosis were suggestive of bacterial infection. The negative CSF Xpert MTB/RIF, blood T-SPOT.TB, tuberculin skin test, and fungal serologies made tuberculous and fungal etiologies less probable. Fever and neurological symptoms resolved within 3 days of antimicrobial therapy, inflammatory markers normalized by day 9, and CSF nucleated cells cleared by day 4. No antiviral therapy was administered. Although CSF viral PCR was not performed, multiple lines of evidence make viral meningitis less likely: first, CRP and PCT levels in the range observed in our patient have been associated with high specificity for bacterial meningitis in published pediatric cohorts, and values in viral meningitis are generally substantially lower; second, persistent neutrophilic predominance in CSF 48 h after symptom onset is a recognized feature favoring bacterial meningitis, while viral meningitis typically shifts to lymphocytic predominance by this time point; finally, the negative CSF culture is consistent with the intravenous ceftriaxone administered 4 h prior to lumbar puncture, which the IDSA guidelines note reduces CSF culture positivity by 50% within 1 h and >80% within 4 h.

Persistent leptomeningeal enhancement is a characteristic neuroimaging finding in infantile bacterial meningitis. Previous studies have reported an overall incidence of 85.0% to 88.0%, and the vast majority of cases resolve gradually within several weeks to 2–3 months after adequate antimicrobial therapy (8, 15–26). In contrast, our patient exhibited persistent enhancement through month 5 without clinical relapse or neurological sequelae, suggesting that this finding may represent prolonged sterile alterations in meningeal microvascular permeability rather than active infection. Furthermore, serial MRI showed no progressive thickening, parenchymal involvement, or new complications, distinguishing this case from previous reports where enhancement was accompanied by leukomalacia or hydrocephalus (27, 28).

The MRI course in this case is exceptionally prolonged. Leptomeningeal enhancement, first detected on treatment day 5, remained essentially unchanged at follow-up examinations on days 15, 65, and 167. Previous studies generally show that isolated leptomeningeal enhancement decreases markedly within 1–8 weeks and resolves within 2 months; persistence beyond 3 months is rare and has been predominantly reported in association with parenchymal complications or arachnoid/dural scarring (8, 29, 30). Our case suggests that isolated leptomeningeal enhancement can persist for more than 5 months despite complete clinical recovery and normalized CSF parameters—a finding rarely reported in pediatric cohorts.

The patient's clinical presentation was consistent with typical infantile CNBM, characterized by fever and nonspecific neurological symptoms such as irritability (16, 17, 19, 22, 24, 31). However, unlike some reported cases, this patient did not exhibit seizures, respiratory failure, or other severe complications (31, 32), nor did the clinical course involve parenchymal brain injury or classic disturbances of consciousness, contrasting with reports of somnolence and poor responsiveness in other cases (17, 22). This milder course may reflect early initiation of appropriate antimicrobial therapy: meropenem plus vancomycin was commenced within 12 h of admission, with adjunctive dexamethasone to reduce meningeal inflammation.

Published data show that the overall treatment success rate for infantile BM is 85.0%–91.0% (8, 15, 18, 26), with mortality rates of 3.0%–8.0% (15, 19, 32). Infants with isolated leptomeningeal enhancement and no parenchymal injury have an excellent prognosis, with only 11.3%–17.0% developing mild developmental delay (15, 20, 25). In contrast, cases complicated by white matter injury or cerebral infarction have sequelae rates as high as 76.0% (8, 15, 17). Our patient's normal growth, neurodevelopmental assessments, and brainstem auditory evoked potentials further support the benign nature of persistent isolated leptomeningeal enhancement. This marks a notable contrast with infections caused by high-risk pathogens such as *Cronobacter sakazakii* or *Elizabethkingia meningoseptica*, which are associated with severe neurological sequelae (16, 17, 31). The patient's congenital secundum atrial septal defect was unrelated to the infection or MRI findings, eliminating underlying structural disease as a confounding factor and supporting the notion that prolonged enhancement itself is non-pathogenic in this context.

The pathophysiology of persistent leptomeningeal enhancement after BM may be explained by a multifactorial “delayed post-inflammatory repair” hypothesis. Basic research has shown that acute neutrophilic infiltration triggers fibroblast proliferation and collagen secretion, leading to meningeal thickening and persistent contrast extravasation (33, 34). We hypothesize that the severe acute inflammation in this case (CRP 116.16 mg/L, 76% CSF neutrophils) initiated a robust fibrotic response. Additionally, developmental differences in fibroblast biology and extracellular matrix remodeling in young infants—including altered matrix metalloproteinase/inhibitor ratios and distinct fibroblast phenotypes—may prolong collagen degradation and tissue remodeling, thereby slowing the resolution of imaging abnormalities (35, 36).

Furthermore, the developing blood-brain barrier (BBB) has structural and functional differences from the mature BBB. While key tight junction proteins are expressed early, endothelial cell-basement membrane-astrocyte end-feet interactions and barrier homeostasis remain immature. This results in slower repair kinetics following inflammatory injury, potentially prolonging contrast extravasation in infants (37, 38). Even after clinical resolution of inflammation, BBB integrity may take months to fully restore, leading to sustained MRI enhancement. While CSF culture remains the diagnostic gold standard for BM, prior antibiotic exposure significantly reduces culture yield, and molecular techniques such as PCR or multiplex panels have higher sensitivity in this setting (39). In our patient, repeated negative CSF cultures, normalized inflammatory markers, and absence of clinical relapse make ongoing active infection or occult biofilm-associated infection unlikely. Nevertheless, guidelines recommend integrating molecular testing with clinical follow-up in diagnostically uncertain cases (12, 40). Additionally, studies have demonstrated that *Escherichia coli* can traverse the BBB via the Lrp-NsrP-PurD pathway (41). Although the causative pathogen was not identified, it is plausible that pathogen-induced meningeal vascular permeability established the pathological basis for persistent enhancement.

This case suggests that persistent leptomeningeal enhancement during recovery from infantile CNBM does not equate to active infection. Imaging changes most likely reflect post-infectious BBB repair and residual inflammatory remodeling. Clinical decision-making should prioritize indicators strongly associated with adverse neurological outcomes, particularly parenchymal injury (white matter lesions, ventriculitis, infarction, abscess), rather than relying solely on meningeal enhancement to assess disease control. This approach may facilitate more precise, rational, and individualized patient management.

Our study has several limitations. First, CSF viral PCR was not performed, so we cannot exclude viral meningitis with certainty. However, the combination of the infant's age, markedly elevated inflammatory markers, neutrophilic CSF pleocytosis, and rapid response to broad-spectrum antibiotics makes a bacterial etiology more probable. Second, CSF pathogen molecular testing (e.g., 16S rRNA PCR) was not performed, so we could not identify the specific causative organism or analyze associations between pathogen type and enhancement duration. Third, this is a single case report, and our findings require validation in larger multicenter cohorts. Finally, follow-up is currently limited to 5 months post-illness; longer-term follow-up is needed to determine the ultimate resolution of enhancement and the infant's long-term neurodevelopmental prognosis.

## 4 Conclusion

Long-term follow-up of this case demonstrates that asymptomatic persistent meningeal enhancement can last for up to five months after clinically diagnosed CNBM in young infants. Critically, this imaging phenomenon may be considered a benign residual finding only after thorough exclusionary diagnosis—specifically, ruling out residual active infection, recurrent infection, other etiologies (e.g., tuberculous or fungal meningitis), and severe neurological complications (e.g., ventriculitis, brain abscess, or parenchymal injury). In the present case, given the compelling but not definitively proven bacterial etiology, such persistent enhancement likely reflects delayed post-inflammatory repair rather than active infection, and was associated with a favorable short-term prognosis as evidenced by the patient's age-appropriate growth and development without neurological sequelae. These findings add to the limited body of evidence on the correlation between imaging manifestations and clinical outcomes in infantile BM. Furthermore, this case highlights the importance of long-term standardized follow-up—which facilitates the exclusion of potential adverse conditions, supports the presumption of benign prognosis, and informs individualized management strategies. Future large-scale cohort studies are warranted to identify risk factors for persistent meningeal enhancement and optimize follow-up intervals, thereby further improving the precision of diagnosis and management in this patient population.

## Data Availability

The original contributions presented in the study are included in the article/Supplementary Material, further inquiries can be directed to the corresponding author/s.
