# Supplementary material for: Persistent meningeal enhancement on MRI in an infant with culture-negative bacterial meningitis: a case report and systematic review of the literature (2014–2025)
Source: Front Pediatr. 2026 Jul 14;14:1799852. doi: 10.3389/fped.2026.1799852 (PMC13410671; doi:10.3389/fped.2026.1799852)
Supplement: Supplementary file 1 [file Supplementaryfile1.docx]

**3 Literature Review**

***3.1 Case Characteristics and Pathogen Distribution***

A systematic search of major English and Chinese databases—including PubMed, Embase, Cochrane Library, Web of Science, CNKI, and Wanfang—was conducted for the period January 2014 to December 2025 using the terms “bacterial meningitis” AND “infant” AND “meningeal enhancement” OR “MRI.” Literature screening and exclusion criteria were as follows: (i) Study subjects aged > 12 months; (ii) Studies related to non-bacterial meningitis (such as viral, tuberculous, and fungal meningitis); (iii) Studies without reporting meningeal enhancement or cranial MRI imaging data; (iv) Secondary studies including reviews and meta-analyses (only primary studies such as cohort studies, case series, and case reports were included); (v) Studies with incomplete data (lacking core information such as age, pathogen, and prognosis) or duplicate publications.

Seventeen studies met inclusion criteria (Supplementary Tables 3), encompassing a total of 754 laboratory-confirmed neonatal and infant bacterial meningitis cases[1-17]. Neonates (0–28 days) accounted for the majority of cases (483/754, 64.1%), including 63 preterm and 420 term infants [1, 2, 4-9, 11, 13]. Infants aged 29 days to 3 months comprised 195 cases (25.9%) [3, 10, 12, 15, 16], and those older than 3 months accounted for 76 cases (10.1%) [11, 14]. Early-onset infection (≤72 hours) occurred in 31.5% of cases [2, 8, 13], whereas late-onset infection (>72 hours) accounted for 68.5% [11, 16, 17]. A clear age–pathogen association was observed: *Streptococcus agalactiae* (group B Streptococcus, GBS) and *Escherichia coli* predominated among infants younger than 59 days; *Streptococcus pneumoniae* accounted for 45% of cases at 60–179 days and 71% beyond 6 months [17].

Pathogen distribution showed Gram-positive organisms accounted for 58.5% of isolates, primarily GBS (25.1%), *S. pneumoniae* (18.9%), and *Staphylococcus epidermidis* (9.0%) [2, 5, 8, 13, 17]. Gram-negative organisms accounted for 41.5%, dominated by *E. coli* (20.8%) and *Serratia marcescens* (4.5%) [1, 2, 12, 13]. High-risk pathogens—including *Cronobacter sakazakii* (4 cases) [4, 9] and *Elizabethkingia meningoseptica* (3 cases) [3]—represented 1.1% and were characterized by multidrug resistance.

***3.2 Initial symptoms and clinical characteristics***

Fever is the most common initial symptom of infantile bacterial meningitis (incidence 82.0%-96.0%) [3, 4, 7-9, 12, 15-17], presenting as high fever (≥39.0℃, 65.0% of cases) or occasional low-grade fever, with apyretic onset (4.0%-13.0%) mainly in premature infants and early-onset infections, characterized by non-specific symptoms like poor response [4, 8, 11]. Neurological manifestations are prominent, including convulsions (28.0%-56.3%) [4, 8, 9, 12, 17], lethargy/poor response (>60.0%)[4, 7, 11, 12, 17], and anterior fontanelle tension/bulging (35.0% in young infants) [15, 16]. Respiratory symptoms (apnea, cyanosis, respiratory failure) occur in 32.0%-45.0% of cases, with premature infants having a higher risk of respiratory failure (29.2%) [11, 17]. Premature infants tend to present with heart rate changes and decreased blood oxygen saturation, while full-term infants are more prone to irritability [11]. Additional non-specific symptoms include feeding refusal, groaning, skin jaundice, diarrhea, and tachycardia [1, 7-9, 15, 16], reflecting the heterogeneous clinical spectrum across age groups.

***3.3 Neuroimaging Findings***

Persistent leptomeningeal enhancement was the most characteristic imaging abnormality, reported in 85.0%–88.0% of cases [2-5, 7, 8, 10, 12, 13, 15-17]. Enhancement patterns included thickened leptomeninges, punctate or curvilinear enhancement, and diffuse meningeal involvement [3, 10, 15]. Reported rates were similar between GBS (49%) and *E. coli* (46%) infections [13]. Synthetic MRI and enhanced FLAIR sequences demonstrated superior sensitivity for detecting subtle enhancement [10, 15].

Complications were variable: ventriculitis occurred in 22.0%–32.0% of cases, with intraventricular purulent material more common in *E. coli* infection (22% vs. 9% in GBS) [12-14]. Brain abscess and subdural empyema occurred in 2.0%–11.0%, with *Cronobacter* infections frequently presenting with multiple abscesses [4, 9, 17]. White matter injury (42.0%), subdural effusion (26.0%–52.0%), and infarction (14.0%–43.0%) were also common [13, 14, 17]. Hydrocephalus occurred predominantly in *E. coli* meningitis (22%) [13]. Only one study proposed a standardized MRI semi-quantitative scoring system comprising 13 items across WMA, GMA, and NPA domains [6].

***3.4 Treatment and Outcomes***

Empirical therapy most commonly included meropenem plus vancomycin or an aminoglycoside combined with a β-lactam, with an overall usage rate of 64% [11] and broad adoption across studies [11, 12, 16]. Targeted therapy was guided by susceptibility testing: penicillin for GBS [5, 17], meropenem or ceftazidime for *E. coli* [1, 13], and specific regimens such as ciprofloxacin plus trimethoprim–sulfamethoxazole for *Elizabethkingia* infections [3]. The typical duration of therapy was 3–4 weeks for uncomplicated meningitis [5, 16] and 6–8 weeks for cases with abscess or empyema [1, 16].

Supportive care included intracranial pressure reduction with mannitol [1, 7, 9], anticonvulsant therapy, and mechanical ventilation (used in 18.0% of cases) [12, 17]. Severe cases required intensive care support, and those with concomitant osteomyelitis or arthritis received adjunctive analgesia or rehabilitation [16]. Treatment success rates ranged from 85.0% to 91.0% [2, 5, 16, 17], whereas mortality ranged from 3.0% to 8.0% [10, 16, 19. *Cronobacter* infections had markedly higher mortality (50%) [4, 9].

***3.5 Prognosis***

MRI abnormalities were strongly associated with long-term outcomes. Patients with isolated meningeal enhancement and no parenchymal injury generally had favorable prognoses, with only 11.3%–17.0% exhibiting mild developmental delay [2, 13, 15]. In contrast, cases with cystic white matter injury, ventriculomegaly, or infarction showed neurodevelopmental delay in up to 76.0% of survivors [2, 4, 17]. Semi-quantitative scoring indicated that preterm infants with WMA ≥3 and term infants with NPA ≥4 had significantly elevated risks of sequelae [6]. MRI abnormalities prompted treatment modification in 45% of cases and extension of antibiotic therapy in 30%, whereas neonates with normal MRI within 7 days had the most favorable outcomes [17].

High-risk pathogens were major determinants of poor prognosis. Survivors of *Cronobacter* and *Elizabethkingia* infections had sequelae rates up to 68.0% [3, 4, 9]. *S. pneumoniae* infection was associated with a 35.0% incidence of hearing impairment [17], and *Pasteurella multocida* infection occasionally progressed to osteomyelitis [16]. Common sequelae included hearing loss (17.0%, predominantly unilateral) [17] and cognitive or motor developmental delay (12.0%–28.5%) [1,6,16]. Some infants, however, exhibited complete recovery following appropriate treatment [10, 15].

Table 3 Summary of meningeal enhancement-related data in 17 studies and the current case

| Study ID | Study type | Total cases | Age group | Meningeal enhancement rate | Duration of meningeal enhancement | Major complications | Key prognostic findings |
| --- | --- | --- | --- | --- | --- | --- | --- |
| 1 | Cohort | 199 | Term neonates | 87.40% | Acute phase only (no long-term follow-up) | Brain infarction, developmental delay | MRI abnormalities correlate with adverse neurological outcomes |
| 2 | Case series | 3 | 2 neonates, 1 35-day-old infant | 100% | Acute phase evolution | Multidrug-resistant *Elizabethkingia meningoseptica* infection, severe sepsis | High risk of poor prognosis |
| 3 | Case report | 1 | 8-day-old neonate | 100% | Acute phase | Multiple brain abscesses | High mortality risk |
| 4 | Case series | 3 | Neonates (7, 16, 23 days) | 100% | Acute phase | Cerebral softening, diffuse brain injury | Poor prognosis |
| 5 | Cohort | 63 | Neonates (18 preterm, 45 term) | 85.70% | Acute phase (semi-quantitative scoring) | Ventriculitis, white matter injury | Higher MRI score predicts worse outcomes |
| 6 | Cohort | 12 | Term neonates | 83.30% | Not specified | Subdural effusion (partial) | Most patients recovered uneventfully |
| 7 | Case series | 10 | Neonates (3 preterm, 7 term) | 90.00% | Acute phase | Sepsis, 1 death | Variable outcomes, severe cases have high mortality |
| 8 | Case report | 1 | 3-day-old neonate | 100% | Acute phase | Subdural empyema | Required surgical intervention |
| 9 | Case series | 20 | Neonates (fatal cases) | 100% | Acute phase | Diffuse cerebral necrosis, multi-organ failure | Universal mortality |
| 10 | Case series | 2 | 39-day-old, 47-day-old infants | 100% | Acute phase | Ventriculitis, cystic encephalomalacia | Severe neurological sequelae |
| 11 | Case report | 1 | 7-week-old female infant | 100% | Short-term follow-up (≤1 month) | Subdural effusion | Required prolonged imaging monitoring |
| 12 | Cohort | 107 | Infants (unspecified) | 47.7% (GBS: 49%, E. coli: 46%) | Acute phase only | Ventriculitis, infarction, hydrocephalus | Pathogen-specific differences in complication rates |
| 13 | Cohort | 44 | Infants <90 days | 86.40% | Acute phase | White matter injury, apnea | Preterm infants have worse outcomes |
| 14 | Cohort | 178 | Infants (0-347 days) | 88.20% | Diagnostic phase only | Infarction, brain abscess | MRI findings influence clinical decision-making |
| 15 | Case report | 1 | 3-month-old male infant | 100% | Not specified | None | No neurological sequelae |
| 16 | Case report | 1 | 5-month-old female infant | 100% | Acute phase | Osteomyelitis | Full recovery |
| 17 | Cohort | 111 | Infants (median 78±79 days) | 57.00% | Hospitalization period | Abscess, infarction, hearing loss | MRI abnormalities indicate more complex treatment |
| **Present case** | **Case report** | **1** | **70-day-old male infant** | **100%** | **~5 months (March–August 2025)** | **None** | **Normal neurodevelopment, no sequelae** |

**Reference**

[1] Li M, Yang H, Tao QJ, Xue YW, Luo R and Cai QY. Neonatal bacterial meningitis with subsequent posterior fossa subdural empyema: a case report. Chinese Journal of Practical Pediatrics 2021; 36: 1428-1430.

[2] Luo XH, Fu YY, Xi YF, Zhang XL, Zhu L, Zeng W and Ju R. Clinical characteristics and pathogen distribution of full-term neonates with bacterial meningitis. Developmental Medicine Electronic Journal 2022; 10: 196-202.

[3] Ren W, Liu L, Xie GQ and Long XL. Clinical analysis of three infants with Elizabethkingia meningoseptica meningitis. Microbes and Infection 2021; 16: 339-342.

[4] Wang P, Zhou W, He J, Tang J, Ouyang S and Chen X. Neonatal Klebsiella pneumoniae meningitis: report of three cases. Chinese Journal of Practical Pediatrics 2018; 033: P.1747-1749.

[5] Yang HH and Li J. Clinical analysis of 12 cases of neonatal Streptococcus agalactiae sepsis with meningitis. Journal of Clinical Pediatrics 2016; 34: 181-184.

[6] Yang MS, Wang L, Zhai Q, Pamir, Zhou J, Cao Y and Qiao ZW. Value of semi-quantitative MRI morphological scoring in predicting discharge outcomes in neonatal bacterial meningitis. Chinese Journal of Evidence-Based Pediatrics 2015; 10: 7.

[7] Yi J, Zhang R and Peng XM. Clinical analysis of neonatal purulent meningitis deaths. Epilepsy and Neurophysiology Journal 2024; 33: 37-43.

[8] Zhao JH, Yang H, Zhou KW and Yu YP. Clinical analysis of 10 neonates with GBS sepsis or meningitis born to GBS-negative mothers. Chinese Journal of Infection Control 2019; 18: 633-637.

[9] Zhou QN, Huang HF, Zhong WH and Shan LQ. A case of neonatal Klebsiella pneumoniae purulent meningitis. Modern Practical Medicine 2019; 31: 1552-1553.

[10] Andica C, Hagiwara A, Nakazawa M, Kumamaru KK, Hori M, Ikeno M, Shimizu T and Aoki S. Synthetic MR Imaging in the Diagnosis of Bacterial Meningitis. Magn Reson Med Sci 2017; 16: 91-92.

[11] Buttera M, Mazzotti S, Zini T, Corso L, Dallai V, Miselli F, Bedetti L, Rossi K, Spaggiari E, Iughetti L, Lugli L and Berardi A. Bacterial Meningitis in Infants Under 90 Days of Age: A Retrospective Single-Center Study. Children-Basel 2024; 11:

[12] Foran AT, Tierradentro-Garcia LO, Haddad S, Martinez-Correa S and Hwang M. Microvascular imaging findings in infants with bacterial meningitis: a case series. J Ultrasound 2024; 27: 911-915.

[13] Kralik SF, Kukreja MK, Paldino MJ, Desai NK and Vallejo JG. Comparison of CSF and MRI Findings among Neonates and Infants with E coli or Group B Streptococcal Meningitis. American Journal of Neuroradiology 2019; 40: 1413-1417.

[14] Kralik SF, Vallejo JG, Kukreja MK, Salman R, Orman G, Huisman T and Desai NK. Diagnostic Accuracy of MRI for Detection of Meningitis in Infants. AJNR Am J Neuroradiol 2022; 43: 1350-1355.

[15] Maruyama S, Kodera K, Kuratuji G and Suda M. [An infant in whom contrast-enhanced fluid attenuated inversion recovery (FLAIR) MRI was useful for the diagnosis of meningitis and devising a treatment strategy]. No To Hattatsu 2017; 49: 42-45.

[16] Nessle CN, Black AK, Farge J and Statler VA. Oligoarticular Hemarthroses and Osteomyelitis Complicating Pasteurella Meningitis in an Infant. Children (Basel) 2017; 4:

[17] Oliveira CR, Morriss MC, Mistrot JG, Cantey JB, Doern CD and Sánchez PJ. Brain magnetic resonance imaging of infants with bacterial meningitis. J Pediatr 2014; 165: 134-139.
